# Ectopic expression of miR156 represses nodulation and causes morphological and developmental changes in *Lotus japonicus*

**DOI:** 10.1007/s00438-014-0931-4

**Published:** 2014-10-08

**Authors:** Ying Wang, Zhishuo Wang, Lisa Amyot, Lining Tian, Ziqin Xu, Margaret Y. Gruber, Abdelali Hannoufa

**Affiliations:** 1 College of Life Sciences, Northwest University, 229 North Taibai Road, Xi’an, 710069 Shaanxi China; 2 Agriculture and Agri-Food Canada, 1391 Sandford Street, London, ON N5V 4T3 Canada; 3 Department of Biology, University of Western Ontario, London, ON N6A 5B7 Canada; 4 Agriculture and Agri-Food Canada, 107 Science Place, Saskatoon, SK S7N 0X2 Canada

**Keywords:** miR156, Plant architecture, Nodulation, Flowering, *Lotus japonicus*

## Abstract

The effects of microRNA156 overexpression on general plant architecture, branching, flowering time and nodulation were investigated in the model legume, *Lotus japonicus*. We cloned an *miR156* homolog, *LjmiR156a*, from *L. japonicus,* and investigated its *SQUAMOSA PROMOTER BINDING PROTEIN LIKE* (*SPL*) genes and its biological function at enhancing vegetative biomass yield, extending flowering time, and its impact on nodulation. Thirteen potential targets for LjmiR156 were identified in vitro and their expression profiles were determined in aerial and underground parts of mature plants, including genes coding for eight SPLs, one WD-40, one RNA-directed DNA polymerase, two transport proteins, and one histidine-phosphotransfer protein. Two SPL and one WD-40 cleavage targets for LjmiR156—TC70253, AU089191, and TC57859—were identified. Transgenic plants with ectopic expression of *LjmiR156a* showed enhanced branching, dramatically delayed flowering, underdeveloped roots, and reduced nodulation. We also examined the transcript levels of key genes involved in nodule organogenesis and infection thread formation to determine the role of miR156 in regulating symbiosis. Overexpression of *LjmiR156a* led to repression of several nodulation genes during the early stages of root development such as three *ENOD* genes, *SymPK, POLLUX, CYCLOPS, Cerberus*, and *Nsp1*, and the stimulation of *NFR1*. Our results show that miR156 regulates vegetative biomass yield, flowering time and nodulation by silencing downstream target *SPLs* and other genes, suggesting that the miR156 regulatory network could be modified in forage legumes (such as alfalfa and trefoils) and in leafy vegetables (like lettuce and spinach) to positively impact economically valuable crop species.

**Electronic supplementary material:**

The online version of this article (doi:10.1007/s00438-014-0931-4) contains supplementary material, which is available to authorized users.

## Introduction

MicroRNAs (miRNAs) have emerged as a new tool to improve plant traits; from biotic and abiotic stress tolerance to grain and biomass yield (Zhou and Luo 2013). Investigating the functions of different miRNAs in fast-growing model plant species may rapidly indicate the range of characteristics that are likely to present themselves when these small RNAs are tested for their economic value at improving slower growing valuable crop plants.

MiRNAs are generally 21–24 nucleotides long non-coding RNA molecules. These are derived from single-stranded RNA precursors that are transcribed by RNA polymerase II, and they can subsequently form an imperfectly matched hairpin structure. In plants, the precursor is further processed by Dicer-like proteins into mature miRNAs which are then incorporated into an RNA-induced silencing complex where they negatively regulate target gene expression at the post-transcriptional level by base-pairing to complementary targets (Dugas and Bartel 2004; Kidner and Martienssen 2005). In some cases, miRNAs can also silence genes at the transcriptional level by affecting chromatin methylation (Brodersen and Voinnet 2009). Temporal and spatial accumulations of a few highly conserved miRNAs are crucial for maintaining proper plant development. For example, *miR165* and *miR166* are involved in the determination of leaf patterns (Liu et al. 2009), *miR156* and *miR157* govern the transition from vegetative to reproductive phase (Wu et al. 2009), and *miR172* participates in controlling floral development (Wollmann et al. 2010). The most conserved miRNAs tend to be the most highly abundant in organisms.

*MiR156*, one of the most conserved and ubiquitous miRNAs in plants, is found in mosses, monocotyledons, and dicotyledons (Arazi et al. 2005; Xie et al. 2006), and has been studied mostly for its role in growth regulation. Although *miR156* and *miR172* function coordinately in plant development, they actually function in opposite ways, with the expression of *miR156* decreasing over time while that of *miR172* increasing (Wu et al. 2009). In *Arabidopsis thaliana*, *Zea mays*, *Oryza sativa*, and *Brassica napus*, overexpression of *miR156* results in dramatic morphological alterations, e.g., dwarf and bushy plants, delayed flowering, varied distribution of trichomes, and increased carotenoid and flavonoid contents (Xie et al. 2012; Hultquist and Dorweiler 2008; Wang et al. 2009; Wei et al. 2010).

In Arabidopsis, 11 out of the 17 members of the *SQUAMOSA PROMOTER BINDING PROTEIN LIKE* (*SPL*) family of genes are targeted by *miR156* (Rhoades et al. 2002; Xing et al. 2010; Gou et al. 2011). MiR156 and its target *SPL*s define an essential regulatory module that controls phase transitions, leaf trichome development, male fertility, embryonic patterning, and anthocyanin biosynthesis (Wang et al. 2009; Yu et al. 2010; Xing et al. 2010; Nodine and Bartel 2010). The *SPL* family encodes plant-specific transcription factors containing at least one SQUAMOSA-PROMOTER BINDING PROTEIN (SBP) domain. In turn, SPLs control the transcription of other genes by binding to promoters that harbor SBP domain-binding sites (SDBs) (Klein et al. 1996). SPL proteins play critical roles in maintaining normal growth throughout a plant’s life cycle. For example, SPL-13 regulates the transition from cotyledons to the appearance of true leaves (Ruth et al. 2010) and SPL-3, SPL-4, and SPL-5 function in determining flowering time through *Flowering Locus D* (FD)-dependent and -independent pathways (Schmid et al. 2003; Wang et al. 2009). Moreover, SPL-3 participates in the regulation of *FLOWERING LOCUS T* (FT) under various ambient temperatures in Arabidopsis (Kim et al. 2012; Hwan Lee et al. 2012). Together with *SPL*-*8*, a gene not targeted by miR156, the miR156-targeted *SPLs* (including *SPL*-*2*, -*9*, and -*15*) are involved in sporogenous cell formation and cell proliferation, all of which influence plant fertility (Xing et al. 2010, 2013). In addition to its role in plant growth and phase transition, the miR156/SPL network coordinates plant development with plant secondary metabolism. For example, overexpression of *miR156* enhances the levels of carotenoids in seeds of *A. thaliana* and *B. napus* (Wei et al. 2010, 2012) and SPL-9 was shown to reduce the production of anthocyanin in *A. thaliana* stems by suppressing the expression of anthocyanin biosynthesis genes (Gou et al. 2011).

WD40-like proteins are also involved in many aspects of plant growth, functioning as a platform for protein–protein and protein–DNA interactions, microtubule organization during mitosis (Zeng et al. 2009), and chromatin conformation (Reyes et al. 2002; Verbsky and Richards 2001). Interestingly, Naya et al. (2010) revealed that a *WD40* transcript is cleaved by *miR156* in the root apices of *Medicago truncatula*. Shi et al. (2005) demonstrated that the Arabidopsis WD-40 protein, SWA1, participates in maintaining root elongation, indicating a potential role for an *miR156/WD*-*40* network in root development.

Legumes are the third largest plant family and are an important source of forage and food. The ability of leguminous plants to interact symbiotically with rhizobia and mycorrhiza makes them useful in maintaining pastures and soil fertility through nitrogen fixation (Graham and Vance 2003). *Lotus*, a genus within Fabaceae, has more than 100 species and some, such as bird’s foot trefoil (*Lotus corniculatus*), have been used as economically important forage crops (Escaray et al. 2012). *Lotus japonicus* is a model plant in legume research due to its relatively small genome, minimal requirements for growing space, and short life cycle (Handberg and Stougaard 1992; Udvardi et al. 2005). Information derived from miRbase (http://www.mirbase.org/, version 20; released in June 2013) revealed 10 and 28 members of miR156 variants in *M. truncatula* and *Glycine max*, respectively, but none in *L. japonicus*.

Aside from their roles in plant development, miRNAs also function in nutrient homeostasis and plant–microbe symbiosis. For example, in *L. japonicus*, miR171 facilitates rhizobial infection while miR397 is activated in nitrogen-fixing nodules (De Luis et al. 2012). Overexpression of *M. truncatula* miR166 reduces the numbers of both nodules and lateral roots (Boualem et al. 2008). MiR169-regulated *MtHAP2*-*1* was shown to play a crucial role in determining the nodule meristematic zone during nodulation in *M. truncatula* (Combier et al. 2006). In *G*. *max*, miR156 and miR166 have five overlapping predicted gene targets (Zeng et al. 2010), which indicates a potential interaction between the two gene systems in root development and nodulation. Both *pre*-*miR156e* and *pre*-*miR156g* are slightly up-regulated in the roots of Arabidopsis when the supply of nitrogen is limited (Pant et al. 2009). Expression of *miR172* is enhanced in soybean roots at 1 and 3 h post-inoculation (hpi) with *Bradyrhizobium japonicum* but returns to basal levels by 12 hpi (Subramanian et al. 2008). In soybean, ectopic expression of miR160 resulted in a decrease in nodulation (Turner et al. 2013). On the other hand, elevated expression of miR482, miR1512, and miR1515 caused increased nodulation in soybean (Li et al. 2010). Opposite roles have been described for miR156 and miR172 in controlling the expression of both symbiotic and non-symbiotic hemoglobins to modulate the extent of nodulation in soybean, with enhanced levels of miR156 being consistent with reduced nodule numbers while miR172 acting as a positive regulator of nodule formation (Yan et al. 2013).

The ability of miR156 to increase shoot branching, delay flowering, and alter secondary metabolism in several plant species (Fu et al. 2012; Gou et al. 2011; Wei et al. 2010, 2012; Wu and Poethig 2006) prompted us to investigate its function in the model forage legume *L. japonicus*. Therefore, we characterized an *L. japonicus* homologue, *LjmiR156a*, focusing on shoot branching, flowering time, and nodulation. Our objective was to investigate the value of the *miR156* gene regulatory system and its potential to improve forage and bioenergy crops where high vegetative biomass production is desirable.

## Materials and methods

### Plant material and growing conditions

*Lotus japonicus* ‘Gifu’ (accession: B-129) was used as the wild-type (WT) germplasm in all experiments. Seeds were germinated in a Petri dish containing eight layers of filter paper soaked in water. After 1 week, the seedlings were transferred into a mixture of vermiculite, sand, and commercial soil (25:25:50) (Premier Tech Horticulture, Rivière-du-Loup, Quebec, Canada). Plants were grown in a chamber at 23 °C, under a 16-h photoperiod and a light intensity of 250 µE s^−1^ m^−2^. Plants were supplemented bi-weekly with a Hoagland nutrient solution.

### Cloning and overexpression of miR156 in *L. japonicus*

Pre-miRNA sequences of *AtmiR156a* (accession: AT2G25095) and *AtmiR156b* (Accession: AT4G30972) from Arabidopsis were used as templates to search for potential *pre*-*miR156* sequences in the *L. japonicus* genome database (http://www.kazusa.or.jp/lotus/). All positive hits were further examined in silico. First, the candidate sequences were tested with a Basic Local Alignment Search Tool (BLAST) through the National Center for Biotechnology Information (NCBI) *Lotus japonicus* EST database (http://blast.ncbi.nlm.nih.gov/Blast.cgi) to find those that could be transcripts. These sequences were then assessed for their capacity to form typical miRNA hairpin secondary structures. Sequences that met these two criteria were selected for further study. This analysis identified a 506-bp cDNA sequence, designated *pre*-*LjmiR156*a, which was amplified by PCR using *LjmiR156a* forward and reverse primers (Supplementary Table S1). The cDNA fragment was cloned into the pJET vector (Thermo Fisher Scientific, Waltham, MA) for sequencing, and then sub-cloned into the pBI121 vector (Jefferson 1987) (replacing the GUS gene) between the *Xba*I and *Sac*I sites. This construct was transformed into *Agrobacterium tumefaciens* strain LBA4404 (Life Technologies, Burlington, Ontario, Canada). Hypocotyls of *L*. *japonicus* were then used for *Agrobacterium*-mediated transformation according to Márquez and Stougaard (2005). Transformants were selected on media containing G-418 (5 µg/ml), and the presence of the transgene in putative transformants was validated by PCR amplification of a partial 35S promoter sequence linked to the *pre*-*LjmiR156a* fragment using *35S promoter* forward and *pre*-*LjmiR156a* reverse primers.

### Small-RNA northern blot analysis

Small RNA was isolated from shoots of mature T_2_ plants using the *mir*Vana miRNA Isolation Kit (Life Technologies) following the manufacturer’s instructions. RNA probes were synthesized with the *mir*Vana miRNA Probe Construction Kit (Life Technologies) using the primers listed in Supplementary Table 1. DynaMarker for small RNA (BioDynamics Laboratory Inc., Hackensack, NJ, USA) was used to determine the target RNA size. Small RNAs were separated on a denaturing 15 % polyacrylamide gel containing 15 % urea in 0.5× Trix/borate/EDTA buffer. After separation on the gel, the nucleotides were transferred onto a nylon membrane and blotted with an RNA probe overnight at 45 °C. The blotted membrane was treated with CDP-Star Chemiluminescent substrate (Sigma-Aldrich, St. Louis, Missouri, USA) and then exposed to X-ray film. The developed films were then scanned by an EPSON V370 scanner and saved as 1,200 dpi Tagged Image Files. The band intensities were then determined by using ImageJ (http://rsbweb.nih.gov/ij/) to measure the mean grey value. The miR156 probe blotted membrane was then stripped by heating the blot in 1 % SDS solution at 85 °C 30 min. The membrane was then blotted with U6 probe which serves as a loading control.

### In silico prediction of Lj-miR156 cleavage targets

The target transcripts for miR156 cleavage were predicted using the psRNATarget web server by following the default parameter (http://plantgrn.noble.org/psRNATarget/). In addition, the mature sequence of *L. japonicus* *miR156* was used to conduct a manual BLAST search against the *L. japonicus* genome database and the NCBI EST database. This eliminated any false candidates that could form potential precursors for *LjmiR156*. After the redundant sequences were manually excluded, potential targets were chosen as candidates for cleavage-site validation using a modified 5′-RACE method (Song et al. 2010). This technique was performed with a FirstChoice RLM-RACE Kit (Life Technologies) according to the manufacturer’s instructions, but with a slight modification. Instead of removing 5′PO_4_, the adaptor was ligated directly to the RNA molecules, which were then subjected to reverse-transcription. Afterward, nested PCRs were run with outer/inner adaptor- and outer/inner gene-specific primers (Supplementary Table 1). The products were gel-purified and cloned into the pJET vector (Thermo Scientific, Waltham, MA, USA). Eleven (11) clones for TC70253, 12 clones for AU089181, and 16 clones for TC57859 were sequenced to determine the cleavage sites of the two candidate genes.

### Quantitative real-time RT-PCR

Specific tissues of *L. japonicus* T_2_ plants and control plants (as mentioned in each experiment) were collected, immediately frozen in liquid nitrogen, and stored at −80 °C for further application. Total RNA was extracted with an RNeasy Plant Mini Kit (QIAGEN, Toronto, Ontario, Canada). The integrity of those samples was examined by running the RNA on a 1 % agarose gel and observing the 18S and 28S bands. This RNA was treated with Turbo DNase I (Ambion, Life Technologies) to eliminate genomic DNA contamination, and was then quantified using a NanoVue (GE Healthcare, Mississauga, Ontario, Canada). Reverse-transcription reactions (1 μg of total RNA per reaction) were performed with a qScript cDNA synthesis Kit (Quanta Biosciences, Gaithersburg, MD, USA) according to the manufacturer’s instructions. QRT-PCR was carried out in a 96-well plate on a C1000 Thermal Cycler and CFX96 Real-Time System (Bio-Rad, Mississauga, Ontario, Canada), with PerfeCTa SYBR Green FastMix (Quanta Biosciences). All primers for qRT-PCR are listed in Supplementary Table 1. Two reference genes—*β*-*ACTIN* (Maeda et al. 2006) and *ATP*-*SYNTHASE* (Andersen et al. 2003)—were used to normalize the data. Transcript levels were calculated based on the ΔΔCT method, with GeneStudy (Bio-Rad).

### Morphological analysis

Seeds from the WT and T_2_ transgenic plants that over-express *LjmiR156a* (*miR156*+) were germinated on filter paper and transferred to soil as described above. Beginning 2 months post-germination, heights and branch numbers were recorded every 2 weeks in the two fertile transgenic seed lines #20 and #22 that were recovered from the transformation of *L. japonicus*. Flowering times were recorded as days post-germination (dpg). Leaves and siliques were photographed under a dissecting microscope, and then measured with ImageJ.

### Nodulation analysis

Surface-sterilized seeds from the two T_2_ seed lines were germinated on eight layers of filter paper soaked in water. At 7 dpg, the seedlings were transferred to vermiculite-filled pots and watered with B&D solution (Broughton and Dilworth 1971) that was supplemented with nitrogen (by adding KNO_3_ to a final concentration of 1 mM). Colonies of *Mesorhizobium loti* harboring the *hemA::lacZ* reporter gene (strain: NZP2235) were cultured at 28 °C for 72 h until OD_600_ = 0.02. The bacteria were washed twice with distilled water followed by centrifugation. They were then re-suspended in water as 1/10 volume of bacterial culture and used for inoculations in which 300 μL were applied to each plant. Afterward, the plants were transferred to a growth chamber and cultured as described above. To evaluate infection threads (ITs), nodule primordia, and nodules, seedlings were removed from the vermiculite at 7, 14, and 21 days post-inoculation. Seedlings were then rinsed with distilled water to remove residual soil. Root-fixation, *LacZ*-staining, and clearing procedures were performed as previously described (Karas et al. 2005). For sample sectioning, fixed samples were embedded into 4 % agarose and sectioned with a Leica microtome VT1000S. The samples were cut into 80-µm sections at a speed of 4.5 and frequency of 70 Hz. The sections were then observed under a Leica stereo dissecting microscope.

### Statistical analysis

Statistically significant differences among treatments were determined with analysis of variance (ANOVA) followed by post hoc Duncan’s test at *P* value ≤0.05.

## Results

### LjmiR156 is highly conserved in plants

To clone *miR156* of *L. japonicus*, we used Arabidopsis pre-*miR156a* and pre-*miR156b* sequences as templates to search the *L. japonicus* genome and EST database. One sequence (GenBank accession: GO023849) was identified and selected for further in silico analysis. This candidate sequence was capable of forming a typical miRNA secondary structure. Because it had the highest homology to *AtmiR156a*, we designated it as *pre*-*LjmiR156a* (Supplementary Fig. S1). Its predicted mature sequence was identical to miR156 from various plants, including *Arabidopsis thaliana* and *Glycine max*. Furthermore, alignments of *pre*-*LjmiR156a* to *pre*-*miR156* sequences from Arabidopsis and *G. max* showed strong similarity not only between the mature miR156 sequences, but also between the *pre*-*mi156* sequences (Supplementary Fig. S2).

### Generating LjmiR156a overexpression plants

**Fig. 1:**
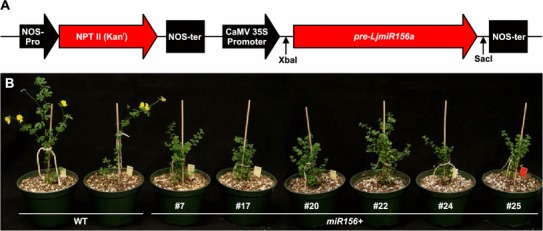
Surviving T_0_ *L. japonicus* plants transformed with *pre*-*LjmiR156a*. **a** Map of *LjmiR156* expression construct pBI121-*LjmiR156a*; **b** phenotypes of *L. japonicus* plants at 2 months after transfer to soil

To investigate the function of *LjmiR156a*, we made a construct for *pre*-*LjmiR156a* (506 bp) for expression under the CaMV35S promoter and used it to transform *L. japonicus* hypocotyl explants (Fig. 1a). After 2 months on B5 medium supplemented with G418 antibiotic, 32 calli were derived from the approximately 150 explants, and of these 19 survived. A total of 17 out of the 19 calli produced shoots after shoot induction and 10 of them regenerated healthy root systems; the presence of the transgene in these plants was confirmed by PCR (Supplementary Fig. S3), and thus they were selected for analysis. Only six transgenic plants survived after transfer to soil, and these plants manifested a dwarfed phenotype, enhanced branching, and flowered late (Fig. 1b). Southern blot analysis indicated that all of these were independent transgenic events (Supplementary Fig. S4). Of the six plants, four were unable to produce enough seeds due to severely delayed flowering, and thus could not be pursued in subsequent generations. The remaining two overexpression plants (lines 20 and 22) maintained a moderate ability to produce seeds and exhibited phenotypes relatively close to those resulting from miR156 overexpression in *A. thaliana* (Wei et al. 2012), *Panicum virgatum* (Fu et al. 2012), and *Solanum lycopersicum* (Zhang et al. 2011).

### Enhanced LjmiR156a expression affects multiple aspects of *L. japonicus* growth

**Fig. 2:**
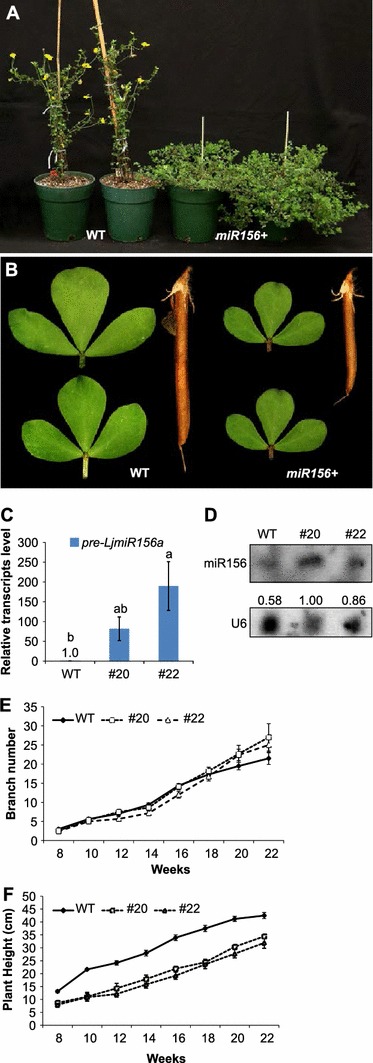
Phenotypic and molecular characterization of transgenic *miR156*+ plants and WT plants. **a** Plant habit of 6-month-old T_0_ transgenic *miR156*+ *L. japonicus* plants. WT (*left*) and *miR156*+ (*right*). **b** Typical leaf and siliques from WT plants and *miR156*+ plants; **c** QRT-PCR showing relative transcript abundance (±SE) of *pre*-*LjmiR156a* in WT and T_2_ miR156+ plants; **d** small-RNA Northern blot on mature miR156 in WT and T_2_ overexpression plants. U6 was used as the loading control. Numbers underneath bands indicate abundance of miR156 when normalized with U6; **e**, **f** comparison of number of shoot branches and plant heights, respectively, between WT and T_2_ *miR156*+ plants

Six months after their transfer to soil, T_0_ transgenic lines 20 and 22 showed greater branching and fewer flowers compared with the ‘Gifu’ WT plants (Fig. 2a). Although the leaf shape was similar between the transgenic plants (*miR156*+) and the WT (Fig. 2b), the surface area was significantly diminished in the former. *MiR156*+ plants also had shorter siliques (Table 1; Fig. 2b), which resulted in substantially fewer seeds per silique. These T_0_ plants were then used in generating T_2_ progeny seed lines for further experimentation to reduce the influence of segregation. Both qRT-PCR on *pre*-*LjmiR156a* and Small-RNA Northern blot analysis on T_2_ plants revealed that the abundance of miR156 in transgenic lines was higher than in WT (Fig. 2c, d).

**Table 1:** Morphological characterization of WT and T_2_ *miR156*+ *L. japonicus* plants

Trait	‘Gifu’ WT	Overexpression line #20	Overexpression line #22
Leaf width (mm)	4.32 ± 0.59	3.37 ± 0.36*	3.50 ± 0.24*
Leaf length (mm)	9.71 ± 0.64	6.51 ± 0.64*	7.09 ± 0.47*
Silique length (mm)	29.10 ± 0.55	21.60 + 0.56*	20.01 ± 0.56*
Flowering time (day)	94.14 ± 3.41	124.57 ± 7.64*	126.43 ± 4.32*

More than five leaves and siliques were randomly collected from each replicate plant. Values are mean ± SE (*n* = 3). The flowering times were recorded from at least five plants from each seed line (values are mean ± SE)

* Significant difference of the means at *P* < 0.05 (one-way ANOVA, Duncan’s test)

In general, all organs from the *miR156*+ transgenic plants were smaller than those from the WT. During the first 3 months, the T_2_ *miR156*+ plants grew slowly, showed a dwarf phenotype, and had fewer branches than the WT (Fig. 2e, f). However, following the WT transition to the reproductive stage (at week 16–18), the transgenic plants gradually out-grew the WT plants and eventually showed an exaggerated branching phenotype. Interestingly, the lateral shoots of *miR156*+ plants appeared early in seedling development, emerging even in axils close to the hypocotyls of 2-month-old seedlings (Supplementary Fig. 5B). Subsequently, the lateral shoots developed vigorously and emerged from almost every leaf axil of *miR156*+, giving those plants an overall bushy phenotype.

### Identification of LjmiR156 targets

**Fig. 3:**
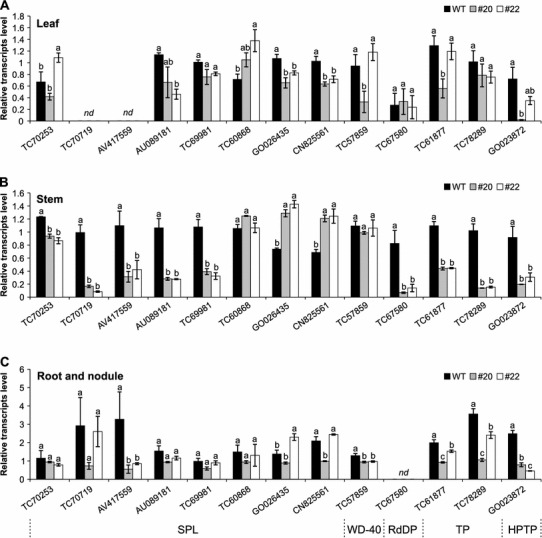
Expression profiles of potential *LjmiR156* target genes (*nd*, not detectable) in leaves (**a**), stems (**b**), and a mixed sample of roots and nodules (**c**). Annotations of candidate genes are shown underneath graph. *RdDP* RNA-dependent DNA polymerase; *TP* trafficking protein; *HPTP* histidine phospho-transfer proteins. Means (±standard error) with the same letter for the same gene indicating no significant difference at *P* ≤ 0.05

**Fig. 4:**
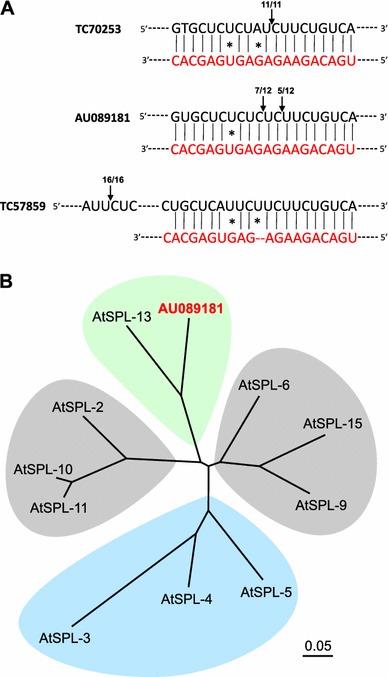
Molecular analysis of *miR156* gene targeting system in *L. japonicus*. **a** Cleavage sites of miR156 in AU089181, TC70253, and TC57859 transcripts as determined by modified 5′-RACE; **b** phylogenetic tree analysis of AU089181 and *AtSPLs*

Based on psRNATarget analysis, we identified 13 candidate target genes of *LjmiR156* in the *L. japonicus* genome (http://www.kazusa.or.jp/lotus/; version 6; released on 18 May 2010) (Table 2). Among them were eight *SPL* genes, one *WD*-*40 like* gene, one predicted RNA-directed DNA polymerase (RdDP) coding gene, one *HISTIDINE*-*PHOSPHO*-*TRANSFER PROTEIN* gene (HPTP), and two genes encoding trafficking proteins (TP). Their expression profiles were further determined in the leaves, stems, and a mixture of roots and nodules (Fig. 3) from T_2_ transgenic lines #20 and #22. Only GO023872 (HPTP) and TC78289 (TP) transcripts were consistently repressed in all three tissue samples of *miR156*+ plants compared to WT plants from all three tissue samples. AV417559 (SPL) and TC61877 (TP) were shown to be down-regulated in *miR156*+ plant stems and underground parts. Interestingly, TC57859, a WD-40 like protein coding gene, was only repressed in the underground parts of *miR156*+ plants. Furthermore, transcript levels of 3 *SPL* candidate genes, TC70253, TC70719, and TC69981, were only decreased in miR156+ plant stems but not in the other two samples. This investigation revealed that transcripts of these target genes were selectively controlled in *miR156*+ lines compared to WT. We then used modified 5′-RACE to validate the miR156 cleavage sites for all 13 candidate genes and we were only able to detect cleavage sites in three of the potential targets, two *SPL*-homologs, AU089181 and TC70253, and one *WD*-*40* homolog TC57859 (Fig. 4a). Phylogenetic tree analysis showed that AU089181 had the highest homology to Arabidopsis *SPL*-*13* (Fig. 4b). TC70253 was not subjected to this analysis because the sequence length was insufficient.

**Table 2:** Candidate genes for Lj-miR156 derived from psRNATarget prediction

Targets (DFCI gene index)	Alignment	Annotation	
TC70253	miRNA 20 CACGAGUGAGAGAAGACAGU 1 Target 133 GUGCUCUCUCUCUUCUGUCA 152	Homologue to SPL protein 13	
TC70719	miRNA 20 CACGAGUGAGAGAAGACAGU 1 Target 155 GUGCUCUCUCUCUUCUGUCA 174	Homologue to SPL	
AV417559	miRNA 20 CACGAGUGAGAGAAGACAGU 1 Target 259 GUGCUCUCUCUCUUCUGUCA 278	Homologue to SPL	
AU089181	miRNA 20 CACGAGUGAGAGAAGACAGU 1 Target 293 GUGCUCUCUAUCUUCUGUCA 312	Homologue to SPL	
TC69981	miRNA 20 CACGAGUGAGAGAAGACAGU 1 Target 1013 GUGCUCUCUAUCUUCUGUCA 1032	Homologue to SPL	
TC57859	miRNA 19 ACGAGUGAGA-GAAGACAGU 1 Target 693 UGCUCAUUCUUCUUCUGUCA 712	Homologue to WD-40	
TC60868	miRNA 20 CACGAGUGAGAGAAGACAGU 1 Target 660 AAGCUCUCUCUCUUCUGUCA 679	Homologue to SPL	
TC67580	miRNA 20 CACGAGUGAGAGAAGACAGU 1 Target 586 GUGCUGACUCUUUUCUGUGA 605	Homologue to RNA-direct DNA polymerase	
TC61877	miRNA 20 CACGAGUGAGAGAAGACAGU 1 Target 843 GUGUUCAAGCUCUUUUGUCA 862	Homologue to transport protein	
TC78289	miRNA 20 CACGAGUGAGAGAAGACAGU 1 Target 291 GUGUUCAAGCUCUUUUGUCA 310	Homologue to transport protein	
GO026435	miRNA 20 CACGAGUGAGAGAAGACAGU 1 Target 444 AUGCUCUCUAUCUUCUGUCA 463	Homologue to SPL	
CN825561	miRNA 20 CACGAGUGAGAGAAGACAGU 1 Target 556 AUGCUCUCUAUCUUCUGUCA 575	Homologue to SPL	
GO023872	miRNA 20 CACGAGUGAGAGAAGACAGU 1 Target 192 GUGGUCACUCUCUUUUUUCA 211	Homologue to histidine phospho-transfer Protein	

Unmatched nucleotides are underlined

### LjmiR156 overexpression affects root development and nodulation

**Fig. 5:**
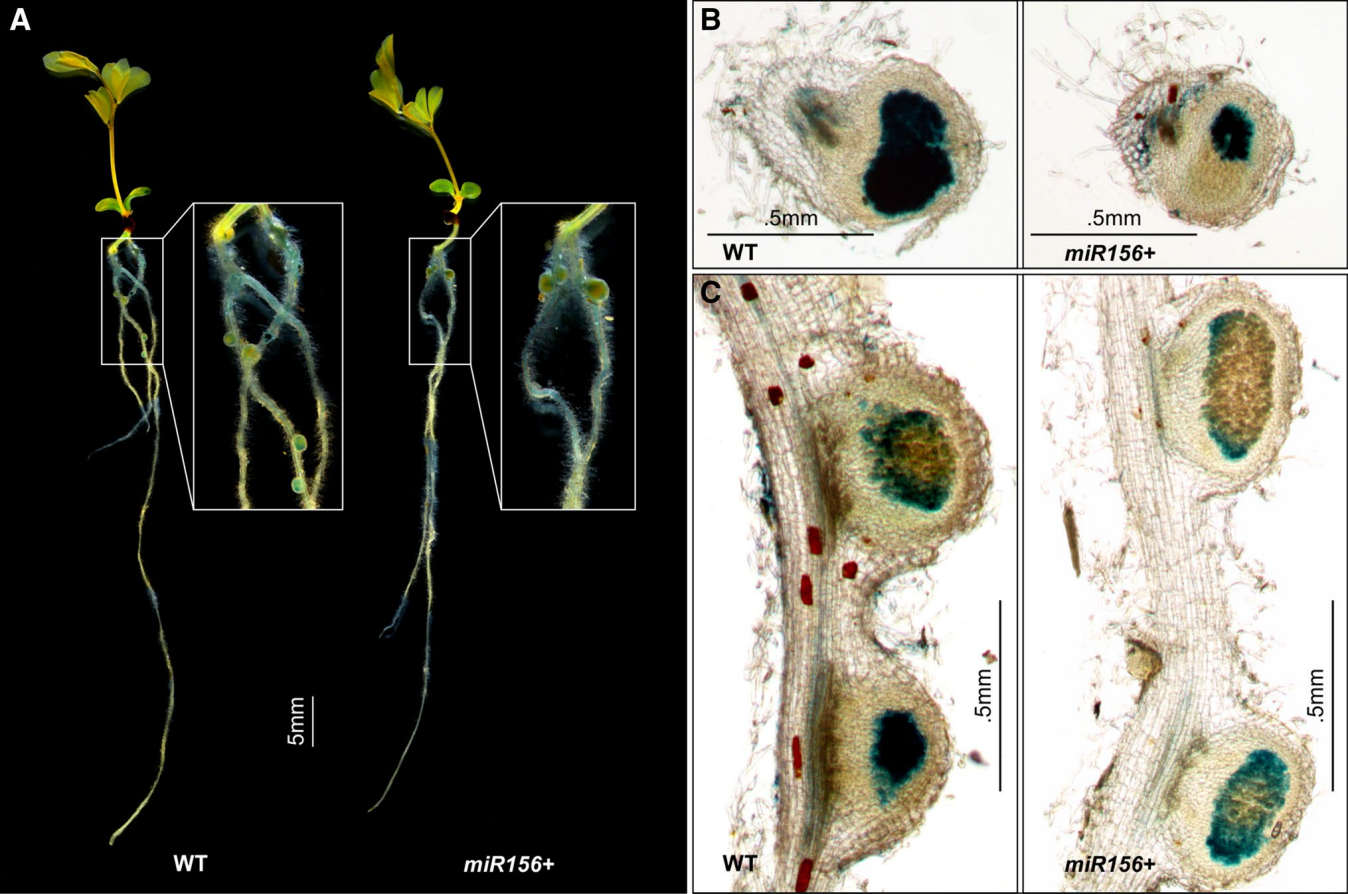
Nodule phenotype of WT and *miR156*+ T_2_ plants 14 days post inoculation (dpi). **a** Whole seedlings of WT (*left panel*) and *miR156*+ plant (*right panel*). Cross sections (**b**) and longitudinal sections (**c**) of nodules from WT and *miR156*+ roots

**Fig. 6:**
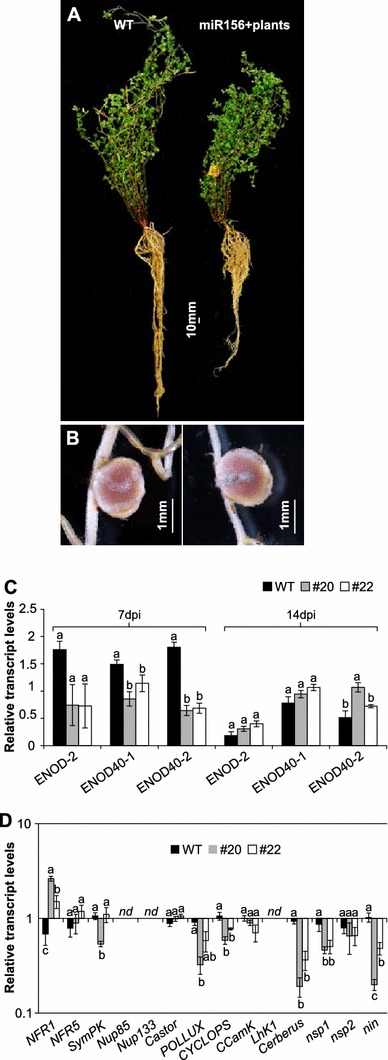
Root phenotype and relative transcript levels of nodulation-related genes in roots (*nd*, not detectable). Means (±standard error) with same letter for the same gene are not significantly different. **a** 4-month-old WT and T_2_ *miR156*+ plants. **b** Mature nodule structures. **c**, **d** Transcript levels of key genes in inoculated roots

To investigate whether Ljmir156 has any effect on *L. japonicus* root growth and symbiosis with rhizobia, we examined root length, nodule number, and the development of infection threads (IT) in T_2_ plants. At 7 dpi with *M. loti*, we observed IT on more than 95 % of the ‘Gifu’ WT roots but on only 10 % of the *miR156*+ roots (Table 3). Roots of the transgenic plants were approximately 15 % shorter and had 50 % fewer nodules than the WT (Table 3). Curiously, we observed that the reduced root development at this early stage of *miR156*+ plants was consistent between *M. loti* inoculated plants and non-bacterial experiment (Table 3). By 14 days, flattened nodules could be seen in miR156+ plants, whereas nodules of WT were more rounded (Fig. 5b, c). By 21 dpi, the number of nodules on the *miR156*+ roots was still approximately 50 % of that in the WT (Table 3; Fig. 5a); however, by this stage there were no detectable differences in nodule morphology (data not shown). After 4 months of growth, the root system of *miR156*+ plants was still underdeveloped compared with the WT, but nodule morphology was the same for both (Fig. 6b). To determine the role of miR156 in regulating symbiosis, we examined the relative transcript levels of several key regulators related to nodulation. At 7 dpi, the transcripts levels of *ENOD40*-*1* and *ENOD40*-*2* were significantly decreased in roots of *miR156*+ plants compared to WT plants (Fig. 6c). Furthermore, 14 key genes involved in IT formation and nodule organogenesis were examined in plant roots at 14 and 21 dpi. At 7 dpi, no significant differences were detected in transcript levels of candidate genes in the roots of WT and *miR156*+ (Supplementary Fig. 6). In contrast, by 14 dpi, expression of *Nfr1* was up-regulated, but *Cerberus*, *CYCLOPS*, *POLLUX, nsp1,* and *Nin* were down-regulated in *miR156*+ plants when compared to WT (Fig. 6d).

**Table 3:** Characterization of symbiosis between WT and *miR156*+ T_2_ *L. japonicus* plants

Time	Morphology	‘Gifu’ WT	*miR156* + #20	*miR156* + #22
7 dpi	No. plants with infection threads	7	2	1
	No. plants with nodule primordia^i^	2	0	0
14 dpi	Root length (cm)	7.24 ± 0.31 (7.28 ± 0.31)	6.19 ± 0.30 * (6.37 ± 0.25)*	5.85 ± 0.26 * (6.08 ± 0.32)*
	Root branch number	2.45 ± 0.26 (2.31 ± 0.26)	1.11 ± 0.19* (1.25 ± 0.29)*	2.27 ± 0.20 (2.23 ± 0.23)
	Nodule number	3.27 ± 0.29	1.67 ± 0.32 *	1.55 ± 0.26*
21 dpi	Root length (cm)	10.49 ± 0.41 (11.57 ± 0.26)	9.64 ± 0.15 * (10.57 ± 0.34)*	9.25 ± 0.46* (10.8 ± 0.35*)
	Root branch number	6.36 ± 0.34 (6.22 ± 0.57)	5.64 ± 0.41* (5.1 ± 0.42)*	5.8 ± 0.55* (7.56 ± 0.69)*
	Nodule number	5.55 ± 0.25	3.82 ± 0.26 *	3.50 ± 0.31*
4 months old	Root length (cm)	43.60 ± 1.86	33.40 ± 1.44*	35.20 ± 1.39*
	Nodule number	432 ± 12.41	288 ± 19.85*	278 ± 17.15*

For 7, 14, and 21 dpi (14-, 21-, and 28-day-old seedlings, respectively) experiment, at least 10 seedlings were collected and measured. Values are mean ± SE. Numbers in brackets were derived from a non-bacterial inoculated control experiment. Root length and nodule numbers from five 4-month-old inoculated seedlings for each line were also counted and measured, respectively. Values are mean ± SE (*n* ≥ 5)

* Significant difference between *miR156*+ and WT plants at *P* < 0.05 (one-way ANOVA, Duncan’s test)

^i^These numbers indicate how many infection threads or nodule primordia were observed out of each 10 plants

## Discussion

Legume forages have an inherent advantage over other forage crops owing to their ability to fix nitrogen (Peter and Graham 2003). Here, we used the model legume *Lotus japonicus* to investigate the role of miR156 in determining legume plant architecture, flowering time, and degree of nodulation. Ectopic expression of *miR156* in *L. japonicus* caused up to a 4-week delay in flowering time in two fertile plants, and most surviving *miR156*+ plants even failed to set flowers by 12 months after tissue culture (the duration of the experiment). The onset of flowering signals a transition from the vegetative to the reproductive stage, and unleashes significant deposition of lignin in the cell walls of some forage plants, which reduces the energy releasable when digesting cell wall structural carbohydrates (Hatfield et al. 2006). Hence, a delay in flowering time would allow for an extended period of vegetative growth, leading to enhanced yield and quality of vegetative biomass. *MiR156*+ plants also showed a two- to three-fold increase in the number of lateral shoot branches since overexpression of miR156 appeared to cause the primary shoots to lose apical dominance. Lateral shoots emerged from almost every axil, and these new lateral shoots also produced secondary laterals. These shoot phenotype changes gave *miR156*+ *L. japonicus* plants an extensive branching phenotype and a bushy appearance. Enhanced miR156 expression in *L. japonicus* also resulted in smaller size organs, including smaller leaves and siliques compared to WT. Other plant species with enhanced miR156 expression generally exhibited similar phenotypes, including enhanced branching, delayed flowering, dwarfed growth, and small leaves. Such phenotypes have been reported as a result of ectopic expression of miR156 in the dicot species, *Arabidopsis thaliana* (Wei et al. 2012)*, Brassica napus* (Wei et al. 2010), *Oryza sativa* (Xie et al. 2012), *Zea mays* (Chuck et al. 2007), *Solanum lycopersicum* (Zhang et al. 2011), and a monocot species *Panicum virgatum* (Fu et al. 2012). The common phenotypic alterations in these diverse species demonstrate that our *LjmiR156* precursor is functional in *L. japonicus*, and suggests that the role of miR156 is highly conserved among different plant species.

We also observed some other important impacts from overexpression of *miR156* in *L. japonicus*. Most of the *miR156*+ plants failed to transition to the flowering stage during the 1-year period of our experiment. Moreover, those that did set flowers (only lines 20 and 22) produced a significantly smaller number of flowers when compared to WT (data not shown). Together with our data, this suggests a role for *LjmiR156* in fertility and flowering through its SPL targets. In Arabidopsis, *SPL*-*8* (a non-*miR156* targeted *SPL*) and miR156-targeted *SPLs* (*SPL*-*2*, *SPL*-*9*, and *SPL*-*15*) function in regulating plant fertility (Xing et al. 2010, 2013). Radically reduced flowering could force plant breeders to rely on vegetative propagation of crops or to rely on conducting seed increases in countries with longer growing seasons, factors which may increase the cost of maintaining and storing varieties. Therefore, from a practical perspective, lines should be selected with only a moderate delay in flowering onset when deploying miR156 in forage breeding.

The root length of *miR156*+ plants was 10–20 % shorter than WT plants, but the roots of *miR156*+ plants had ~50 % fewer nodules than the WT *L. japonicus* plants indicating that ectopic expression of *LjmiR156* affects plant symbiosis with rhizobia. To confirm this phenomenon was not caused by rhizobia directly, a non-bacterial inoculation experiment was carried out parallel to the inoculation experiment. Here, we observed that the differences in root length and branching patterns between *miR156*+ and WT plants were consistent between these two conditions. So we believe that the impacts on root system were caused by miR156 rather than by rhizobia. Symbiosis with soil organisms has always been a valuable attribute for maintaining soil fertility and reducing fertilizer input. In other plants, functional characterization of miR156 focused mainly on above-ground traits, i.e., shoot branching, flowering, and seed composition (Fu et al. 2012; Wei et al. 2010; Wu and Poethig 2006). Here, we demonstrated the role for *LjmiR156a* in the symbiotic relationship between *L. japonicus* and rhizobia. The impact of this reduction in nodulation should now be examined in greater detail in *miR156*+ plants to determine whether resources (e.g., carbohydrates) were diverted away from symbiotic rhizobia to allocate more energy toward developing vegetative biomass.

To further elucidate the relationship between miR156 and nodulation, we examined 14 nodule organogenesis-related genes in roots of *miR156*+ and WT plants at 7 and 14 dpi. The genes we tested have been shown earlier to affect many aspects of *L. japonicus* nodule development (Madsen et al. 2010), including *Nfr1* and *Nfr5* (for nod-factor perception); *SymRK*, *Nup85*, *Nup133*, *Castor*, and *POLLUX* (for signal transduction); *Cyclops* and *CCaMK* (for Ca^2+^ signal interpretation); *Cerberus* (potential protein degradation); *Lhk1* (for cytokinin signaling); and *Nsp1*, *Nsp2*, *and Nin* (for transcriptional regulation). At 7 dpi, *Lhk1* and *Nin* were down-regulated while *Nup133*, *POLLUX*, and *CCamK* were slightly up-regulated. At 14 dpi, only *NFR1* was up-regulated, while *POLLUX*, *CYCLOPS*, *Cerberus*, *nsp1*, and *Nin* were down-regulated. Within these five genes, *NIN* has been shown to be involved in both infection thread formation and nodule organogenesis. As a nodulation-specific transcription factor, NIN is associated with the development of root nodule primordia (Schauser et al. 1999; Marsh et al. 2007), and its loss-of-function was shown earlier to inhibit root nodule organogenesis (Soyano et al. 2013).

Nodules of WT and *miR156*+ *L. japonicus* plants were morphologically similar at mature nodule stages (but had a flattened phenotype early on), while *miR156*+ plants had reduced numbers of infection threads and nodule primordia. Reduced transcription of the five key symbiotic genes above at 14 dpi, but not at 7 dpi suggests that the reduced nodule number in the transgenic plants might not be directly related to these regulators at the transcriptional level. On the other hand, three *ENOD* genes were down-regulated at 7 dpi in the *miR156*+ plants relative to the WT controls, with more than a twofold reduction observed for *ENOD40*-*2* in both transgenic lines. These early nodulin (*ENOD*) genes were shown earlier to be induced during nodulation in *L. japonicus* (Takeda et al. 2005). Hence, we hypothesize that miR156 may function at the early stages of nodule biogenesis by directly or indirectly targeting *ENOD40* genes. In the future, the three SPL genes that were down-regulated in the roots of *miR156*+ plants, AU089181, AV417559, and TC70253, could be tested to determine whether they are the direct link between *miR156* and *Nin*, *ENOD2*, *ENOD40*-*1* and -*2*. The fact that miR156 expression is induced under conditions of nitrogen deficiency (Pant et al. 2009) indicates a possible role in nutrient homeostasis, especially nitrogen fixation through plant–microbe symbiosis. A recent study in soybean showed that overexpression of *miR172* enhanced nodule numbers, whereas elevated *miR156* expression repressed nodule formation (Yan et al. 2013). This is consistent with our findings on reduced nodulation in *miR156*+ *L. japonicus* plants. Taken together, our results suggest that overexpression of *LjmiR156* inhibits nodule formation, and that *miR156* temporally regulates nodulation-related genes.

Several plant hormones, including auxin and gibberellins can positively or negatively regulate nodulation (Maekawa et al. 2009; Deinum et al. 2012). These hormones function at different stages in the nodulation process and may participate in the coordination of epidermal and cortical cell development (Ding and Oldroyd 2009). The recently discovered phytohormone, strigolactone which is derived from carotenoids, has a positive impact on nodulation in *Pisum sativum* (Foo and Davies 2011) and inhibits shoot branching downstream of auxin in Arabidopsis (Brewer et al. 2009). Our previous results showed that miR156 affects carotenoid accumulation in *B. napus* and Arabidopsis (Wei et al. 2010; 2012). Given that strigolactones are carotenoid catabolism products and that changes were observed in nodulation and shoot branching in *miR156*+ *L. japonicus* plants, strigolactones should now be tested in this species to determine whether they are impacted (directly or indirectly) by miR156 and the SPL regulatory system.

In summary, our data clearly show that *LjmiR156* controls a network of downstream genes likely through the silencing of its target *SPL*s in the model legume *L. japonicus*, and that this system in turn regulates the expression of other downstream developmental and quality trait genes. Thus, by fine-tuning the control of this regulatory network, it may be possible to target specific desirable aspects of plant growth and development. For example, SPL-9 regulates the expression of *TCL1* and *TRY*, which determine trichome density and distribution in Arabidopsis (Yu et al. 2010). As well, SPL-9 regulates dihydroflavonol reductase which is involved in flavonoid biosynthesis in Arabidopsis (Gou et al. 2011). Some quality traits, e.g., increased branching, delayed flowering, and higher biomass yield, are highly sought after by the forage industry; hence, our findings in *L. japonicus* are highly applicable to the world’s major forage crops, such as *Medicago sativa* (alfalfa). As with other species, we anticipate that different target genes alone (or possibly in combination) will affect only one or a limited number of plant traits. Once their downstream target genes are identified and characterized, it may be possible to influence only desirable traits by manipulating the expression of specific target genes rather than the entire miR156 gene regulatory network. In the future, we intend to increase the level of miR156 genes in forage crops and leaf vegetable crops where extended vegetative growth is highly desirable. We will also investigate the role of its downstream targets to determine which genes can be selectively targeted to improve crop yield and quality with little or no impact on root growth and nodulation.

## Electronic supplementary material

Below is the link to the electronic supplementary material.

Supplementary material 1 (PDF 566 kb)
