# Supplementary material for: Ectopic expression of miR156 represses nodulation and causes morphological and developmental changes in Lotus japonicus
Source: Mol Genet Genomics. 2014 Oct 8;290(2):471–84. doi: 10.1007/s00438-014-0931-4 (PMC4361721; doi:10.1007/s00438-014-0931-4)
Supplement: Supplementary file 1 — Supplementary material 1 (PDF 566 kb) [file 438_2014_931_MOESM1_ESM.pdf]

Supplementary Files:

Supplementary Table 1. Primers used in this study.

Gene (Accession number)	Usage	Forward primer (5’-3’)	Reverse primer (5’-3’)
LjmiR156a	Gene cloing	TCTAGAGCAAACCCTAATAAAATCTCCATA	GAGCTCAATAAGCGCCAAAGCAACAGTAG
NPTII	Probe synthesis	GAGCACGTACTCGGATGGAAG	GTAAAGCACGAGGAAGCGGTC
B-actin	qPCR	GCATTGTTGGTCGTCCTCGT	TGTGCCTCATCCCCAACATA
ATP-synthase	qPCR	CAATGTCGCCAAGGCCCATGGTG	AACACCACTCTCGATCATTTCTCTG
TC70253	qPCR	CATCACAGACTCACGCACCT	GGCTCCATAAGGGACATTGA
TC70719	qPCR	CCTTCAATGTTCCCCTTTCA	TAGTCCTCATGGCCTTGTCC
AV417559	qPCR	CAATGGCCAATATCCACCTT	AATTCCTGACGATTGGCTTG
AU089181	qPCR	TCGAATCGACAAGCACAGAC	CTATTGCCCTTTCCACTTGC
TC69981	qPCR	CTCTCTTCAGACGGCAAACC	CCGAAATCCAAAGATCCTGA
TC57859	qPCR	ATGCTGTGTTGCTTGCTCAC	ATCCCAGTCAAACCATCTGC
TC60868	qPCR	GAAGAAAAGGGTGGTGGTGA	GCCGCTTAGATCAGCATAGC
TC67580	qPCR	AAGGGTGAATCGTTGGTGAG	CTACAGGGGTTGGACTTGGA
TC61877	qPCR	GGCTTTCACGGATGTCAGTT	AGATGCATGCTCATTGCTTG
TC78289	qPCR	GCAGGTTCATTTGTGGTGTG	GAAATATTCCCCGGTCCAGT
GO026435	qPCR	GCGGCATAAGGTTTGTGAGT	CTGCTACATTGCTGGCAAAA
CN825561	qPCR	GCGGCATAAGGTTTGTGAGT	CTGCTACATTGCTGGCAAAA
GO023872	qPCR	AAGCAACCCAGATTTTGTGG	TGGAGCTGCTACCCTTCAGT
Nfr1 **(AJ575249)**	qPCR	CACAGAACCGCAGGTCTAGC	CTGCACTACTAGAGGCATTACCA

***Nfr5* (AJ575255)**	qPCR	CACTGCTGCAACCAACCTTC	AGTTAGCAACGTGCATCCCA
***SymPK* (AF492655)**	qPCR	ACCTCCTGCAGTTTACAGGC	CTCTGCCTTGCCAACAACAC
***Nup85* (AB284835)**	qPCR	GCGGTTTACCCTCTCAACCA	GCAGAAGGTTCGGCGAAAAG
***Nup133* (AJ890251)**	qPCR	AATTGAAGGAGCTGAAGAGCAGA	GGTCCCAAACTTTCACGCATAAT
***Castor* (AB162157)**	qPCR	TGGCCTTGACATAAGTCGGT	AGGGAGACGCCTAGCTTGTA
***POLLUX* (AB162158)**	qPCR	GGAAATGCTGTCATTAGGCG	GACGTCTTGACTGTATATCACGGA
***CYCLOPS* (EF569221)**	qPCR	TGGCTAACAAATGGAGAGGGAT	CCATCTGAGGCTACACCAACTT
***CCamK* (AM230793)**	qPCR	TGTGGAGGTGCTGAAAGCAA	CCTTGGTGATGCACCCTGAT
***Cerberus* (AB505797)**	qPCR	TTGGTGTCAGCATTTGAGTTGT	TCCTGCCATCTTAGCTCCTT
***nsp1* (EF012819)**	qPCR	GGAAGGGGCAACCTGTTTCT	TGTACAGAACTCCCACCCCA
***nsp2* (DQ665943)**	qPCR	GGGCAACCCTTCTCATTCCA	GCAACTGAATTAGGCGAGCG
***nin* (AJ239041)**	qPCR	TCCAATGCTCTTGATCAGGCT	ACCACACTTGCCTTGTTGGA
***Lhk1* (DQ848999)**	qPCR	CCAGTTGAAGTTTCACTCGACG	AGCAAACCTTATAGGATGGAGACC
ENOD2	qPCR	GTGGCATGCATGTTTTGGTTA	GCAGAAGTGGAGTATTGTACT
ENOD40-1	qPCR	CCTCTGAACCAATCCATCAAATCCA	GTGGAGGAGTGTGAGAGGTGACAGC
ENOD40-2	qPCR	GTCGCACTTGCAGTTGTGGATC	GACTTGCCGGTTCGCCAGGCTG
miR156	Probe synthesis	TGACAGAAGAGAGTGAGCACCCTGTCTC	

U6	Probe synthesis	TCATCCTTGCGCAGGGGCCACCCTGTCTC
**TC70253**	5’RACE Outer Primer	GGTCACTCCCACTATGAACCAAGACT
**TC70253**	5’ RACE Inner Primer	ATTCATTCTACCCCATTCCATCCA
**TC57859**	5’RACE Outer Primer	TGAGCCTTCTTTATCGTTGTCTGA
**TC57859**	5’ RACE Inner Primer	TGCATCCGCTAAAGAGTTCACGA

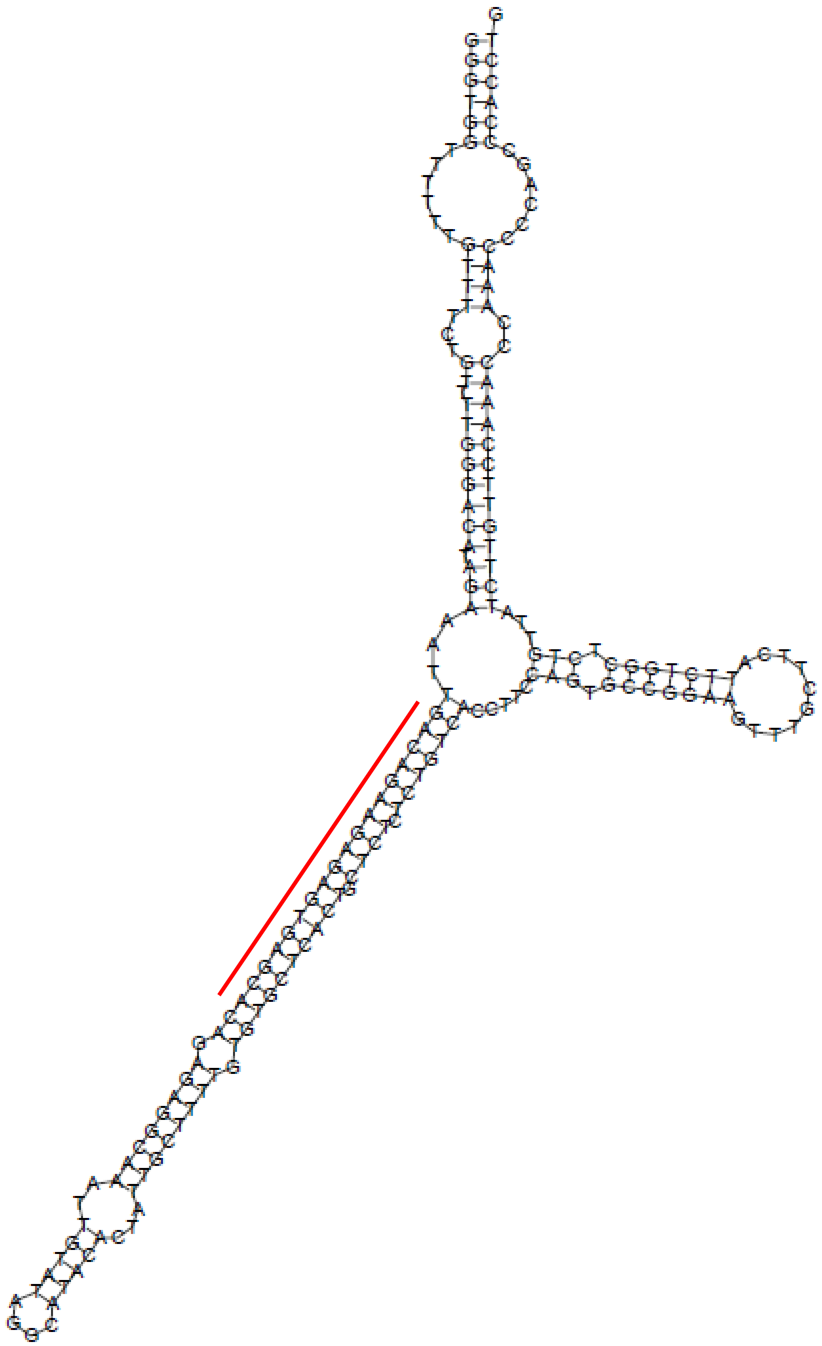

Supplementary Fig. 1. Secondary structure of *pre-LjmiR156a*. Mature miRNA is indicated by red bar. Mature miRNA and miRNA* are indicated by underlines.

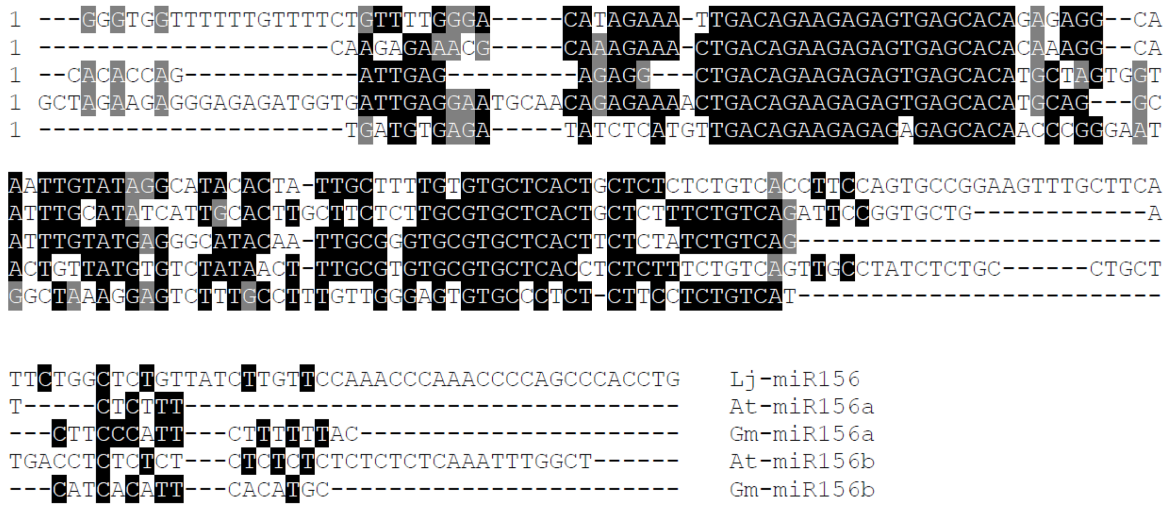

Supplementary Fig. 2. Sequence alignment of *pre-LjmiR156a* and *pre-miR156* from *Arabidopsis thaliana* and *Glycine max*.

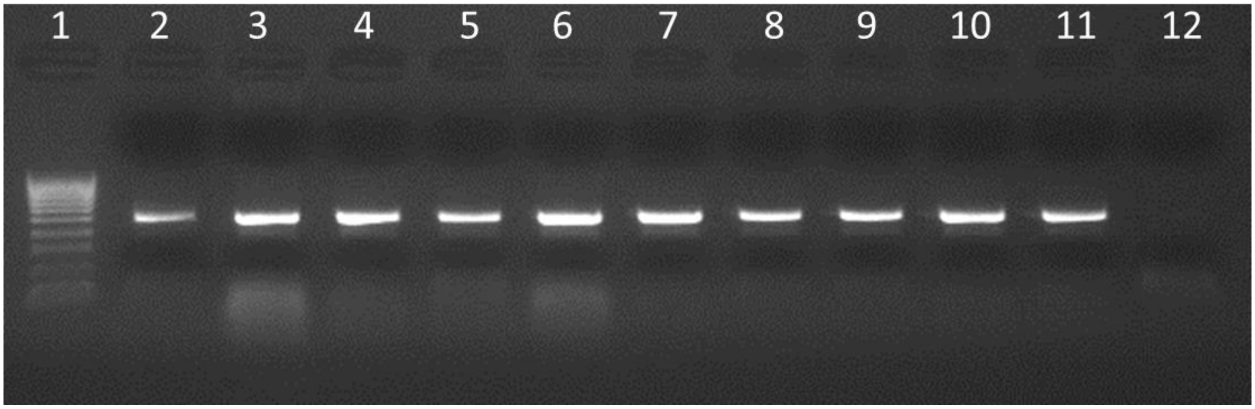

Supplementary Fig. 3. Validation of transgenes by PCR.

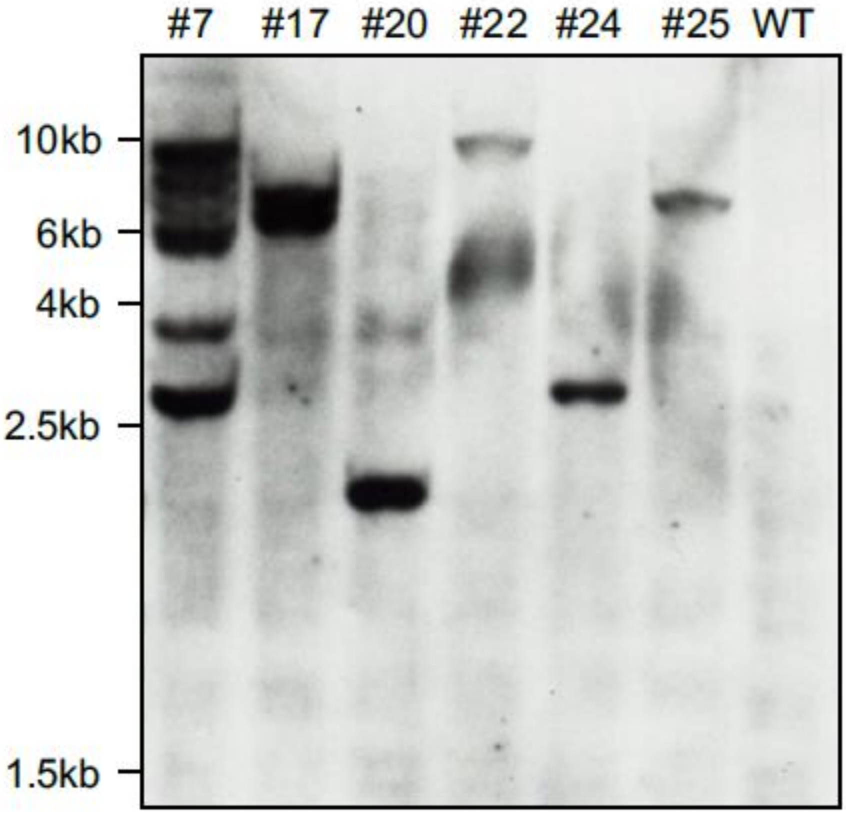

Supplementary Fig. 4. Southern blot analysis of transgenic plants. Genomic DNA was isolated by CTAB method [Rogers SO BA (1988) Extraction of DNA from plant tissues. In: Gelvin SB SR (ed) Plant molecular biology manual, vol A6. Kluwer, Dordrecht, pp 1-10]. Briefly, approximately 15 μg of genomic DNA was digested overnight with *Eco*R I (Fermentas), separated on a 0.8% agarose gel, and transferred to a nylon membrane. As a probe, a 305-bp *NPTII* fragment was amplified from plasmid pBI121 with forward primer NPTII_F and reverse primer NPTII_R (Supplementary Table 1). The NPTII probes were labelled with digoxigenin (DIG), using a PCR DIG Probe Synthesis Kit (Roche). Hybridization (ULTRAhyb Hybridization Buffer; Ambion) and detection (CDP-Star; Roche) were performed according to the manufacturer’s instructions.

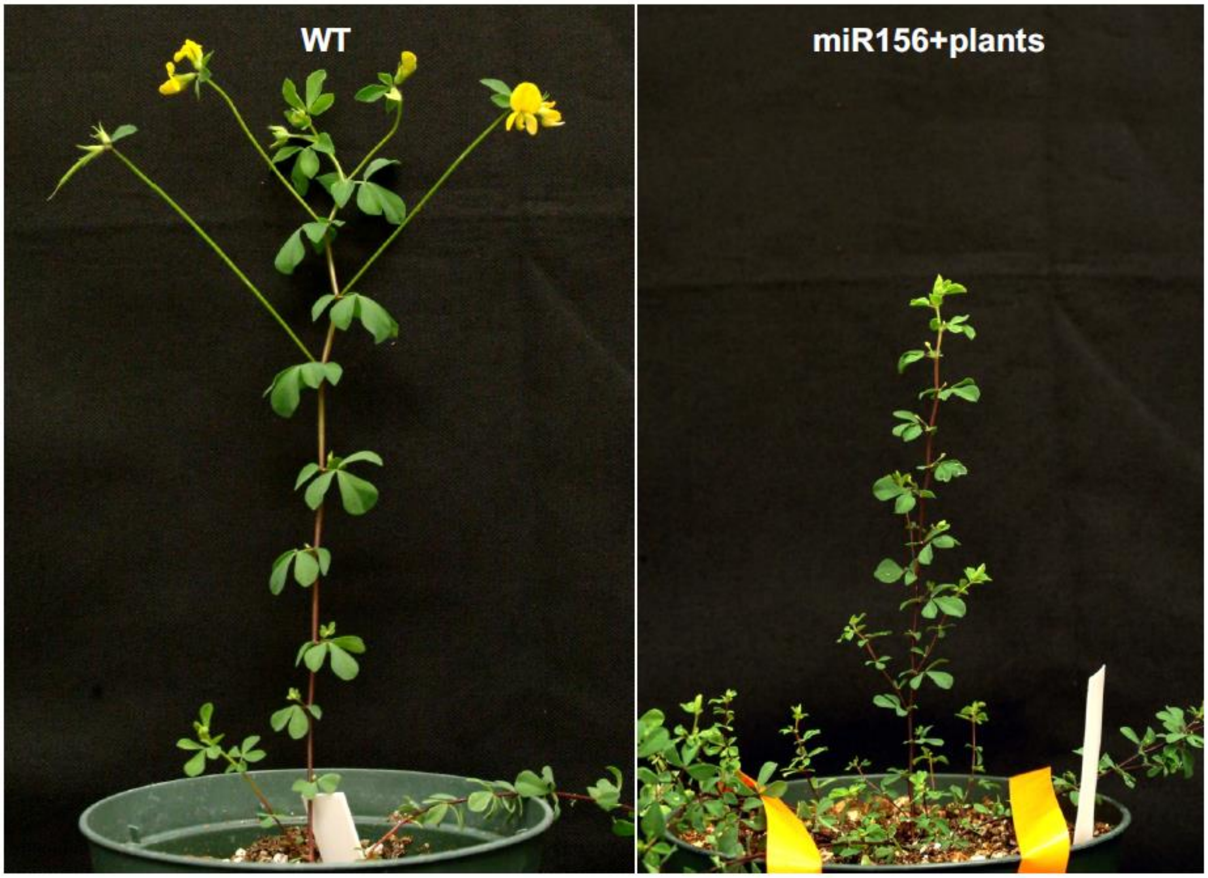

Supplementary Fig. 5. More secondary branches in miR156+ plants compared with WT plants.

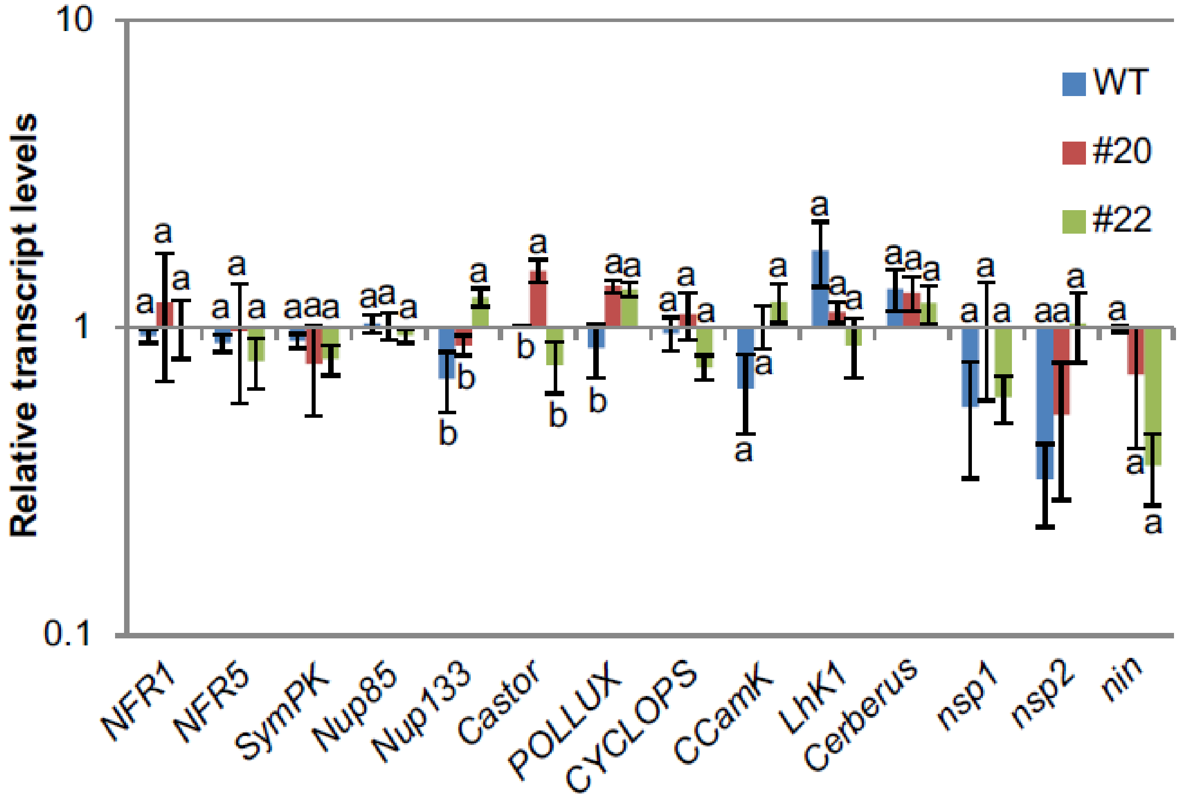

Supplementary Fig. 6. Transcripts levels of nodulation related genes at 7 dpi stage. Means (± standard error) with the same letter for the same gene indicating no significant difference at P≤0.05.
